# Performance of iCare quantitative computed tomography in bone mineral density assessment of the hip and vertebral bodies in European spine phantom

**DOI:** 10.1186/s13018-023-04174-w

**Published:** 2023-10-16

**Authors:** Feng Liu, Hongmei Zhu, Jinlian Ma, Liqiong Miao, Shuang Chen, Zijie Yin, Huan Wang

**Affiliations:** Department of Medical Imaging, Jiangyin Hospital Affiliated to Nanjing University of Chinese Medicine, 130 Renmin Zhong Lu, Jiangyin City, 214400 Jiangsu Province China

**Keywords:** Osteoporosis, Quantitative computed tomography, Osteodensitometry, European spine phantom, Hip fracture

## Abstract

**Background:**

Osteoporosis is a systemic bone disease which can increase the risk of osteoporotic fractures. Dual-energy X-ray absorptiometry (DXA) is considered as the clinical standard for diagnosing osteoporosis by detecting the bone mineral density (BMD) in patients, but it has flaws in distinguishing between calcification and other degenerative diseases, thus leading to inaccurate BMD levels in subjects. Mindways quantitative computed tomography (Mindways QCT) is a classical QCT system. Similar to DXA, Mindways QCT can directly present the density of trabecular bone, vascular or tissue calcification; therefore, it is more accurate and sensitive than DXA and has been widely applied in clinic to evaluate osteoporosis. iCare QCT osteodensitometry was a new phantom-based QCT system, recently developed by iCare Inc. (China). It has been gradually applied in clinic by its superiority of taking 3-dimensional BMD of bone and converting BMD values to T value automatically. This study aimed at evaluating the osteoporosis detection rate of iCare QCT, compared with synchronous Mindways QCT (USA).

**Methods:**

In this study, 131 patients who underwent hip phantom-based CT scan were included. Bone mineral density (BMD) of the unified region of interests (ROI) defined at the European spine phantom (ESP, German QRM) including L1 (low), L2 (medium), and L3 (high) vertebral bodies was detected for QCT quality control and horizontal calibration. Every ESP scan were taken for 10 times, and the mean BMD values measured by iCare QCT and Mindways QCT were compared. Hip CT scan was conducted with ESP as calibration individually. T-scores gained from iCare QCT and Mindways QCT were analyzed with Pearson correlation test. The detection rates of osteoporosis were compared between iCare QCT and Mindways QCT. The unified region of interests (ROI) was delineated in the QCT software.

**Results:**

The results showed that there was no significant difference between iCare QCT and Mindways QCT in the evaluation of L1, L2, and L3 vertebrae bodies in ESP. A strong correlation between iCare QCT and Mindways QCT in the assessment of hip T-score was found. It was illustrated that iCare QCT had a higher detection rate of osteoporosis with the assessment of hip T-score than Mindways QCT did. In patients < 50 years subgroup, the detection rate of osteoporosis with iCare QCT and Mindways QCT was equal. In patients ≥ 50 years subgroup, the detection rate of osteoporosis with iCare QCT (35/92, 38.0%) was higher than that with Mindways QCT. In female subgroup, the detection rate of osteoporosis with iCare QCT was significantly higher than Mindways QCT. In male subgroup, the detection rate of osteoporosis with iCare QCT was also markedly higher than Mindways QCT. The detection rate of osteoporosis by iCare QCT was higher than Mindways QCT with hip bone assessment. Of course, the results of the present study remain to be further verified by multicenter studies in the future.

## Background

Osteoporosis, a systemic bone disease, is featured by low bone mass and bone microstructure destruction, which can increase the risk of osteoporotic fractures [1, 2]. Osteoporotic fracture is a severe disease among the elderly and seriously affects the quality of life [3]. It is estimated that more than 200 million women suffer from osteoporosis globally, and about 30% women and 20% men aged over 50 experience osteoporosis-related fractures [4]. Osteoporotic fractures occur most frequently in hip, wrist, humerus, and spine, with estimated new cases of 9 million osteoporotic fractures each year [5]. Osteoporotic fractures elevate the short-term mortality rate, as well as long-term disability rate [6]. Hip fractures are regarded as the most destructive type of osteoporotic fractures for the association with high mortality and disability rate, as well as increased economic burden [7]. It is estimated that global hip fractures will reach 4.5 million in 2050 from 1.26 million at 1990, and half of the hip fractures are possibly to happen in Asia, especially in China [7]. Therefore, early diagnosis and prompt treatment of osteoporosis are critical.

Since the onset of osteoporosis is hidden, screening of osteoporosis in high-risk population is particularly important [8]. Clinical diagnosis for osteoporosis requires tools that precisely evaluate the mineral content of the bone [9]. Bone mineral density (BMD) measurement is the main basis for screening and diagnosing osteoporosis [10]. Since 1994, the standard diagnostic technique for osteoporosis has been dual-energy X-ray absorptiometry (DXA), which was proposed by World Health Organization (WHO) [11]. DXA is a simple, convenient, and low-radiation technique to evaluate BMD; however, DXA could not provide 3-dimensional (3D) imaging of the bone microstructure, for the projected region analyzed by DXA includes both cortical bone and trabecular bone, thus resulting in the underdiagnosis of osteoporosis [12]. Moreover, degenerative changes are often unidentified in DXA images in posterior anterior projection, leading to overestimated BMD values [13].

Quantitative computed tomography (QCT), which was initially introduced by Harry Genant and Douglas Boyd in 1977 [14], has become an alternative technique of DXA to determine BMD [12]. QCT is accepted to detect and monitor osteoporosis over time [15]. Similar to DXA, QCT also measures the attenuated radiation of the subject with X-rays, but synthesizes cross-sectional images [12]. QCT can directly present the density of trabecular bone without overlying cortical bone, and vascular or tissue calcification [16]; therefore, QCT is more accurate and sensitive than DXA in the evaluation of osteoporosis [11]. Although QCT is still less frequently used in clinic than DXA, previous studies have reported that QCT possessed a higher osteoporosis detection rate than DXA did [3, 6, 17]. Thus, QCT is a promising technique for the assessment of osteoporosis.

There are two calibration strategies for QCT analysis: phantom-based calibration and phantom-less calibration [18]. In the phantom-based calibration, a phantom is needed to provide reference values, which is comprised of different concentrations of calcium hydroxyapatite compartments [15]. The phantom could be used simultaneously with patients or separately in a series of assessments [15]. In the phantom-less calibration, patient’s own tissues were used as internal calibration references [15]. However, the evidence of the application of internal calibration is insufficient [19]. Therefore, phantom-less calibration is less frequently used in clinic than phantom-based calibration.

Mindways QCT (Mindways Inc., USA) is a reliable system for BMD evaluation, and it is widely applied in clinic [20–22]. So Mindways QCT has been used to assess the capacities of new ways to evaluate BMD [9, 15, 23–25]. iCare QCT osteodensitometry is a new phantom-based QCT system, recently developed by iCare Inc. (China), which is economic and has been gradually applied in clinic. This study aimed at evaluating the osteoporosis detection rate of iCare QCT, compared with synchronous Mindways QCT based on the European spine phantom.

## Materials and methods

### Patients

A total of 131 patients who underwent hip CT scan between January 2023 and March 2023 at Jiangyin Hospital Affiliated to Nanjing University of Chinese Medicine were enrolled. Population baseline characteristics are summarized as followed: mean age, 59 ± 15.82 years; gender, 67/64 (F/M); and BMD range: 165.802–509.316 mg/cm^3^. This study has been approved by the ethics committee of Jiangyin Hospital Affiliated to Nanjing University of Chinese Medicine and consistent with Helsinki Declaration.

Inclusion criteria: (1) completion of hip CT imaging; (2) aged 18–70 years; (3) informed consent has been obtained. Exclusion criteria: (1) tumor, fracture or tuberculosis at the hip; (2) history of chronic diseases that may affect bone metabolism, including kidney diseases, thyroid diseases, parathyroid diseases, adrenal diseases, diabetes, and malignant tumors; (3) history of medication that may affect bone metabolism, such as hormones, fluoride, and bisphosphonates.

### Equipments

#### Osteodensitometry

iCare (ICare Inc., China) QCT osteodensitometry and Mindways (Mindways Inc., USA) QCT osteodensitometry were both performed on each participant with ESP phantom calibration, respectively.

#### Phantoms

European spine phantom (ESP, German QRM) was applied in the present study, with main body composed of water-equivalent resin. The ESP consists of three types of inserts in the trabecular compartments with varying amounts of hydroxyapatite (low density, 50 mg/cm^3^; medium density, 102 mg/cm^3^; and high density, 197 mg/cm^3^) for the 1st lumbar vertebrae (L1), the 2nd lumbar vertebrae (L2), and the 3rd lumbar vertebrae (L3), respectively. ESP scans were taken for 10 times, and both iCare and Mindways were employed to measure BMD of the L1, L2, and L3 vertebral bodies in ESP, individually. The mean values of BMD at L1, L2, and L3 in ESP by iCare QCT and Mindways QCT were compared, respectively, as well as the corresponding errors of accuracy.

#### CT scan technique

This study employed a 32-detector CT scanner (SIEMENS, SOMATOM go.Up, Germany). The CT scan parameters included a voltage of 120 kV, a current of 250 mA, a thickness of 5 mm, a matrix of 512 × 512, and a field of view of 40 cm. And all the CT examinations were conducted without contrast agent. Each subject was placed in supine position with ESP under the hip region. The lower limbs of each subject were fixed, and CT scans on bilateral hips were performed from the apex of the femoral head to 3 cm below the lesser trochanter of the femur.

### BMD assessment and diagnostic criteria

The CT imaging data were transferred to the iCare QCT and Mindways QCT workstations, respectively. Two experienced physicians conducted the measurement. The unified region of interests (ROI) was delineated in the QCT software, and BMD of the cancellous bone of the hip joint was measured. Values of the hip BMD were automatically converted to T-scores in the QCT system. If BMD ≤ 2.5 SD T-score, osteoporosis was diagnosed [26]. SD is the standard deviation of peak BMD of a young normal reference population [26].

### Statistical analysis

Statistical analyses were conducted by SPSS 17.0 software. The numeric data were expressed as mean ± standard deviation, and analyzed by paired t test. Pearson correlation analysis was applied for the correlation analysis between iCare QCT and Mindways QCT with hip T-scores with SPSS 17.0 [27]. The comparisons of osteoporosis detection rates were evaluated by Chi-square test with SPSS 17.0 [28]. *P* < 0.05 indicated significant difference.

## Results

### Performance of iCare QCT and Mindways QCT in BMD evaluation of L1, L2, L3 vertebral bodies of ESP

Mean BMD values and standard deviation for L1, L2, and L3 measured by iCare QCT and Mindways QCT are presented in Table 1. These data indicated that both iCare QCT and Mindways QCT could efficiently reflect the BMD of ESP, and there was no significant difference in the evaluation of ESP between iCare QCT and Mindways QCT (iCare QCT vs Mindways QCT: L1, *P* = 0.335; L2, *P* = 0.709; L3, *P* = 0.796).

**Table 1:** BMD of L1, L2, L3 vertebral bodies of ESP evaluated by iCare QCT and Mindways QCT

	n	L1 (50)	L2 (102)	L3 (197)
iCare QCT	10	53.38 ± 5.61	109.44 ± 12.42	206.25 ± 22.47
Mindways QCT	10	51.77 ± 0.69	108.08 ± 1.1	204.55 ± 1.61
*t*		0.986	0.378	0.261
P		0.335	0.709	0.796

### Pearson correlation analysis of T-scores of the hip assessed by iCare QCT and Mindways QCT

Pearson correlation was applied to evaluate the correlation of the hip T-scores assessed by iCare QCT and Mindways QCT (*r* = 0.836, *P* = 0.000). There results indicated that there was a strong correlation of iCare QCT and Mindways QCT in the assessment of hip bone mass.

### The detection rates of osteoporosis by evaluation of hip T-score with iCare QCT and Mindways QCT

Hip BMD values were automatically converted to T-scores in the QCT system. As described in material and methods, if BMD <  − 2.5 SD T-score, osteoporosis was diagnosed. Table 2 shows the detection rates of osteoporosis by evaluating the hip T-score with iCare QCT and Mindways QCT. Of the 131 included patients, 38 (29.0%) patients were identified as osteoporosis by iCare QCT, while 28 (21.4%) patients identified as osteoporosis by Mindways QCT (*X*^2^ = 62.833, *P* = 0.000). These results indicated that iCare QCT had a higher detection rate of osteoporosis with the assessment of hip T-score than Mindways QCT.

**Table 2:** Detection rates of osteoporosis by evaluation of hip T-score with iCare QCT and Mindways QCT

Methods	Number of the patients	Detection rate of osteoporosis
iCare QCT	131	38 (29.0%)
Mindways QCT	131	28 (21.4%)
*X*^2^		62.833
P		0.000

### Subgroup analysis for age

Table 3 shows the subgroup analysis for age with the detection rates of osteoporosis by the evaluation of hip T-score with iCare QCT and Mindways QCT. In patients < 50 years subgroup, the detection rate of osteoporosis with iCare QCT and Mindways QCT were equal (7.7%). In patients ≥ 50 years subgroup, the detection rate of osteoporosis with iCare QCT (38.0%) was higher than that of Mindways QCT (27.2%, *X*^2^ = 42.404, *P* = 0.000). These results indicated that iCare QCT is more effective than Mindways QCT in the assessment hip T-score in aged population.

**Table 3:** Detection rates of osteoporosis by the evaluation of hip T-score with iCare QCT and Mindways QCT in patients in different age subgroups

Methods	Age	Number	Detection rate	*X*^2^	*P*
iCare QCT	< 50	39	3 (7.7%)	–	–
Mindways QCT	< 50	39	3 (7.7%)		
iCare QCT	≥ 50	92	35 (38.0%)	42.404	0.000
Mindways QCT	≥ 50	92	25 (27.2%)		

### Subgroup analysis for sex

Table 4 shows the subgroup analysis for sex with the detection rates of osteoporosis by the evaluation of hip T-score with iCare QCT and Mindways QCT. In female subgroup, the detection rate of osteoporosis with iCare QCT was significantly higher than Mindways QCT (*X*^2^ = 33.120, *P* = 0.000). In male subgroup, the detection rate of osteoporosis with iCare QCT was also markedly higher than Mindways QCT (*X*^2^ = 28.070, *P* = 0.000). These results indicated that iCare QCT had higher detection rates of osteoporosis with hip T-score in both male and female population than Mindways QCT.

**Table 4:** Detection rate of osteoporosis by evaluation of hip T-score with iCare QCT and Mindways QCT in gender subgroups

Methods	Sex	Number	Detection rate	*X*^2^	P
iCare QCT	Female	67	24 (35.8%)	33.120	0.000
Mindways QCT	Female	67	21 (31.3%)		
iCare QCT P	Male	64	14 (21.9%)	28.070	0.000
Mindways QCT	Male	64	7 (10.9%)		

## Discussion

This study evaluated the detection capacity of osteoporosis by iCare QCT, compared with Mindways QCT. Both iCare QCT and Mindways QCT could efficiently reflect the bone density at the L1 (low), L2 (medium), and L3 (high) vertebrae bodies of ESP; iCare QCT and Mindways QCT had a strong correlation in the assessment of hip T-score; iCare QCT had higher detection rate of osteoporosis than Mindways QCT with hip T-score analysis.

Mindways QCT is a classical QCT system, and has been widely applied in clinic to evaluate osteoporosis. Mindways QCT could be used in the detection of osteoporosis at the hip, vertebrae body, and peripheral skeleton [29]. Several previously published studies applied Mindways QCT as control to evaluate the efficacy of different brands of QCT or ways for the osteoporosis assessment [9, 15, 23–25]. Zhao et al. applied Mindways QCT system as standard control to evaluate the efficacy of five brands of QCT for the evaluation of vBMD [9]. Therkildsen et al. applied Mindways QCT system as contrast to assess phantom-less QCT for the evaluation of bone mass [15]. Ziemlewicz et al. applied Mindways QCT system to compare unenhanced and contrast-enhanced CT to evaluate proximal femur BMD for osteoporosis screening [23]. In this study, Mindways QCT was applied as well to evaluate the detection rate of osteoporosis by iCare QCT.

It is a common sense that hip fracture is a serious type of fracture, and the one-year mortality rate of hip fracture reaches 22–40% [7, 30]. Fifty percent patients after hip fracture experience functional independence lost, and one in three ultimately becomes fully dependent [7]. The etiologies of hip fracture are complex, including low BMD, aging, female sex, etc., while the main risk factor of hip fractures is the decrease in BMD [31]. With the rapidly expanded aging population world widely, the socioeconomic burden of hip fracture is becoming a great challenge. Thus, identifying high-risk individuals for hip fracture is a pertinent objective. Although the detection of osteoporosis by aBMD with DXA has been regarded as clinical standard to evaluate osteoporosis, DXA measures aBMD with a projectional image, which could not differentiate calcification and other degenerative changes, thus resulting in an overestimation of BMD [32]. The predictive performance of hip fracture by aBMD was moderate with area under the receiver operating characteristic curve (AUC) around 0.80 [33]. The reasons for the moderate predictive capacity of aBMD may be both the function of factors that cannot be measured by DXA, including bone microstructure (bone mineral properties and composition) and macroarchitectural factors (bone shape, and 3D vBMD, and cortical thickness) [33]. QCT is superior to DXA for the evaluation of BMD since it can reveal the 3D micro and macro architectures of the bone, as well as cortical bone thickness [33]. It has been reported that vBMD is more strongly associated with fracture risk than aBMD by DXA [34, 35]. Recently, Carballido-Gamio et al. developed a multi-parametric model based on QCT, which could efficiently predict hip fracture [33]. Therefore, QCT is gradually applied in clinic and technically renewed over time. In the current study, the osteoporosis detection capacity of the newly developed iCare QCT system was assessed.

The ESP (QRM, Germany) is a widely applied semi-anthropomorphic phantom that could be both used in QCT and DXA [9]. The ESP is made up of water-equivalent resin, which contains three simulated vertebrae bodies with different contents of hydroxyapatite that represent vertebrae bodies with low, medium, and high bone mass [9]. The assessment of the vertebrae bodies of EPS could reveal the accuracy of QCT. In general, before running the QCT software, quality control (QC) must be conducted [36]. Firstly, calibration phantom was scanned with the identical protocol in both ESP and patient scans [36]. Secondly, QC calibration was conducted with QCT software with the known BMD inserts in the ESP [36]. QC calibration could determine the relationship between BMD and CT values, which can be applied to convert the following CT values into BMD [36]. Zhao et al. found a significant difference in the assessment of the low, medium, and high density vertebrae bodies of the EPS in five brands of QCT system [9]. In the current study, ESP (QRM, Germany) with both iCare QCT and Mindways QCT was scanned, and then, T-scores were transformed with the calculated BMD values automatically in the QCT system. Both iCare QCT and Mindways QCT could effectively reveal the bone contents of L1 (low), L2 (medium), and L3 (high) vertebrae bodies of the ESP. And there was no significant difference between iCare QCT and Mindways QCT for the evaluation of L1, L2, and L3 vertebrae bodies of EPS.

BMD is the recommended standard index for the diagnosis of osteoporosis. In the evaluation of the hip bone mass, the values of the hip BMD by QCT are transformed into T-scores to assess osteoporosis [32]. T-score ≥  − 1.0 is defined as normal, ≤ 1.0 and ≥ 2.5 as osteopenia, and ≤  − 2.5 as osteoporosis [37]. In addition, Z-scores can be used in premenopausal female, male less than 50 years old, and children, with Z-score ≤  − 2.0 considered to be lower than the expected normal range [32]. In this study, BMD evaluated by iCare QCT and Mindways QCT was both transformed automatically to T-score in the QCT system. T-scores evaluated by iCare QCT and Mindways QCT were strongly correlated, but the detection rate of osteoporosis with iCare QCT was significantly higher than Mindways QCT did (29.0% vs 21.4%).

The morbidity of osteoporosis is increasing with age, especially in those over 50 years old [32]. Bone synthesis and resorption are regulated by the activity of osteoblasts and osteoclasts, and a number of molecules participated in the process of bone metabolism, including estrogen, calcitonin, vitamin D, parathyroid hormone, etc. [38]. Bone synthesis is predominant in young people, but since the third decade, trabecular bone mass begins to decrease, and over sixty, for the decrease of hormone levels, the cortical bone mass begins to decrease as well [38]. Aging in female patients is related to a negative balance in remodeling, with increased bone remodeling in cancellous and cortical bone and decreased trabecular thickness, resulting in the loss of bone mass and bone microarchitecture disruption [10]. While in male patients, aging is mainly related to the decreased bone formation and bone turnover [10]. In the current study, 39 patients (39/131, 29.8%) were less than 50 years old, while 92 patients (92/131, 70.2%) over 50 years old. Of the 39 patients < 50 years, only 3 patients (7.7%) were diagnosed osteoporosis by both iCare QCT and Mindways QCT. In subgroup over 50 years old, the detection rate of osteoporosis by iCare QCT was 38.0% (35/92), and by Mindways QCT was 27.2% (25/92). The rate of osteoporosis in the present study was significantly higher in ≥ 50 years old subgroup than < 50 years old subgroup, which was in accordance with previously published studies [4, 10]. Moreover, iCare QCT presented a significantly higher detection rate of osteoporosis than Mindways QCT with the assessment of hip bone mass in patients over 50 years old. But in patients less than 50 years old, the detection rate of osteoporosis was the same by iCare QCT and Mindways QCT (7.7%), which was partly because of the low morbidity rate of osteoporosis in patients less than 50 years old.

Previously studies have reported that the prevalence of osteoporosis differed in sex [10, 39, 40]. A recent study reported that osteoporosis was detected in 29.1% females and 6.5% males over 50 years old in China, which was estimated in population prevalence as 49.3 million women and 10.9 million men, respectively [41]. Postmenopausal females are five to ten times more vulnerable to osteoporosis than male [6]. The main reasons for the difference in prevalence of osteoporosis between women and men mainly include lower bone mass in females than in males, calcium consumption during pregnancy, and most importantly, decreased level of estrogen in older age [42, 43]. In this study, 67 (67/131, 51.1%) female patients and 64 (64/131, 48.9%) male patients were included. Of the 67 female patients, the detection rate of osteoporosis by iCare QCT (24/67, 35.8%) was significantly higher than that of Mindways QCT (21/67, 31.3%). Of the 64 male patients, the detection rate of osteoporosis by iCare QCT (14/64, 21.9%) was also markedly higher than that of Mindways QCT (7/64, 10.9%). The detection rate of osteoporosis by both iCare QCT and Mindways QCT revealed a higher prevalence of osteoporosis in females, which was consistent with the previously published studies [10, 39, 40]. And in both females and males, iCare QCT presented a higher detection rate of osteoporosis Mindways QCT.

The strength of the current study is that the detection rate of osteoporosis assessed by iCare QCT was evaluated, compared with Mindways QCT. iCare QCT presented a higher detection rate of osteoporosis than Mindways QCT in the evaluation of hip bone mass. However, there also existed some limitations. The present study is a single-center study, and the conclusions from the present study need further evaluation in multicenter studies. In addition, only the hip bone mass was evaluated, bone mass at other sites of the body, such as spine and peripheral bones may need further evaluation.

## Conclusion

The detection rate of osteoporosis by iCare QCT was higher than Mindways QCT with hip bone assessment, and no significant difference was found in evaluating L1, L2, and L3 vertebrae bodies of ESP. Of course, the results of the present study remain to be further verified by multicenter studies in the future.

## Data Availability

Data and material described in this study are available from the authors upon reasonable request and availability.
